# 2023/24 mid-season influenza and Omicron XBB.1.5 vaccine effectiveness estimates from the Canadian Sentinel Practitioner Surveillance Network (SPSN)

**DOI:** 10.2807/1560-7917.ES.2024.29.7.2400076

**Published:** 2024-02-15

**Authors:** Danuta M Skowronski, Yuping Zhan, Samantha E Kaweski, Suzana Sabaiduc, Ayisha Khalid, Romy Olsha, Sara Carazo, James A Dickinson, Richard G Mather, Hugues Charest, Agatha N Jassem, Inès Levade, Maan Hasso, Nathan Zelyas, Ruimin Gao, Nathalie Bastien

**Affiliations:** 1 British Columbia Centre for Disease Control, Vancouver, Canada; 2 University of British Columbia, Vancouver, Canada; 3 Public Health Ontario, Toronto, Canada; 4 Institut National de Santé Publique du Québec, Québec, Canada; 5 University of Calgary, Calgary, Canada; 6 Queen’s University, Kingston, Canada; 7 Public Health Laboratory, Alberta Precision Laboratories, Edmonton, Canada; 8 National Microbiology Laboratory, Public Health Agency of Canada, Winnipeg, Canada

**Keywords:** Influenza, SARS-Co-V-2, Omicron, XBB.1.5, vaccine effectiveness, test-negative design, observational study

## Abstract

The Canadian Sentinel Practitioner Surveillance Network reports mid-season 2023/24 influenza vaccine effectiveness (VE) of 63% (95% CI: 51–72) against influenza A(H1N1)pdm09, lower for clade 5a.2a.1 (56%; 95% CI: 33–71) than clade 5a.2a (67%; 95% CI: 48–80), and lowest against influenza A(H3N2) (40%; 95% CI: 5–61). The Omicron XBB.1.5 vaccine protected comparably well, with VE of 47% (95% CI: 21–65) against medically attended COVID-19, higher among people reporting a prior confirmed SARS-CoV-2 infection at 67% (95% CI: 28–85).

The 2023/24 influenza season in Canada has been characterised by influenza A(H1N1)pdm09 predominance with co-circulation of influenza A(H3N2) and severe acute respiratory syndrome coronavirus 2 (SARS-CoV-2) [1,2]. The Canadian Sentinel Practitioner Surveillance Network (SPSN) reports mid-season 2023/24 vaccine effectiveness (VE) against both influenza and COVID-19, including detailed genetic characterisation of contributing viruses in relation to respective vaccine strains.

## Epidemiological context

The SPSN enrolled consenting patients with acute respiratory illness (ARI), including new or worsening cough, who presented within 7 days of illness onset to designated community-based sentinel practitioners in Alberta, British Columbia (BC), Ontario and Quebec. The test-negative case–control design was used to estimate VE against medically attended outpatient ARI due to laboratory-confirmed influenza or SARS-CoV-2 [3]. For comparison with historical SPSN analyses, influenza VE was also explored among those presenting with influenza-like illness (ILI), requiring cough but additionally fever and one or more of sore throat, arthralgia, myalgia or prostration; fever was not required among patients ≥ 65 years. Mid-season analysis included specimens collected between 29 October 2023 (week 44) and 13 January 2024 (week 2), tested at accredited provincial laboratories by real-time RT-PCR and/or multiplex assays.

Updated 2023/24 multivalent influenza and monovalent SARS-CoV-2 Omicron XBB.1.5 vaccines became available in Canada through universal publicly funded immunisation campaigns beginning October 2023, with their co-administration encouraged [4]. Of publicly funded influenza vaccines in SPSN provinces, > 98% were egg-based inactivated products. Compared with 2022/23, the 2023/24 influenza A(H1N1)pdm09 vaccine component was updated to A/Victoria/4897/2022-like (clade 6B.1A.5a.2a.1, hereafter written as 5a.2a.1); the A(H3N2) component remained unchanged as A/Darwin/9/2021-like (clade 3C.2a1b.2a.2a, hereafter written as 2a) [5]. Community-dwelling adults ≥ 65 years in all provinces (except Quebec ≥ 75 years) were administered MF59-adjuvanted (BC, Ontario) or high-dose influenza vaccines (Alberta, Ontario, Quebec). The SARS-CoV-2 Omicron XBB.1.5 vaccines included two mRNAs and one adjuvanted recombinant product, with a recommended interval of at least 3–6 months since receipt of the last non-XBB.1.5 COVID-19 vaccine (6 months preferred) [4].

Influenza vaccine status was predicated on report by the participant or guardian via the sentinel practitioner, as per usual SPSN practice [6]. Provincial immunisation registries for COVID-19 were considered more complete than for influenza and more reliable than self-report. XBB.1.5 VE estimation was therefore restricted to provinces where these immunisation registries could be used (BC, Ontario and Quebec). Children < 12 years were excluded from XBB.1.5 VE analyses owing to more complex dosing requirements [4]. Primary XBB.1.5 VE analyses excluded recipients of non-XBB.1.5 vaccine within 6 months before the launch of the provincial XBB.1.5 immunisation campaign. We also explored this in sensitivity analyses excluding those who had received non-XBB.1.5 vaccine within 3 months before campaign launch and alternatively without any such exclusion applied. We also explored XBB.1.5 VE among participants who reported (or whose guardians reported) any SARS-CoV-2 infection at any time prior to the current illness that was confirmed by nucleic acid amplification test (NAAT) or rapid antigen test (RAT), the latter including self-administered testing. Whereas in primary analyses an influenza test-positive case could contribute as a SARS-CoV-2 test-negative control (and vice versa), to explore bias due to the possible indirect confounding pathway of correlated influenza and COVID-19 vaccination, we excluded influenza cases from SARS-CoV-2 controls (and vice versa) [7]. Finally, to address sparse data issues, we applied Firth’s method of penalised logistic regression or modified covariate categories, as indicated [8-10].

## Virological characterisation

Whole genome sequencing (WGS) of influenza viruses was undertaken on original participant specimens by Canada’s National Microbiology Laboratory [11]. Where WGS did not meet minimum inclusion criteria for haemagglutinin (HA) sequences as specified in Supplementary Table S2, Sanger sequencing was additionally undertaken. The HA clades were assigned as per Nextclade [12], specifying additional amino acid substitutions (beyond those that were clade-defining) and affected antigenic sites in parentheses, and annotating involvement of the receptor binding site (RBS) or gain/loss of glycosylation (e.g. +/− CHO). Influenza vaccine reference sequences accessed through the Global Initiative on Sharing All Influenza Data (GISAID) are acknowledged in Supplementary Table S1. Whole genome sequencing of SARS-CoV-2 case viruses from original patient specimens was undertaken per routine provincial or national laboratory protocols using contemporary Pango nomenclature [13], with other details provided in Supplementary Table S3.

## Virological findings

**Figure 1:**
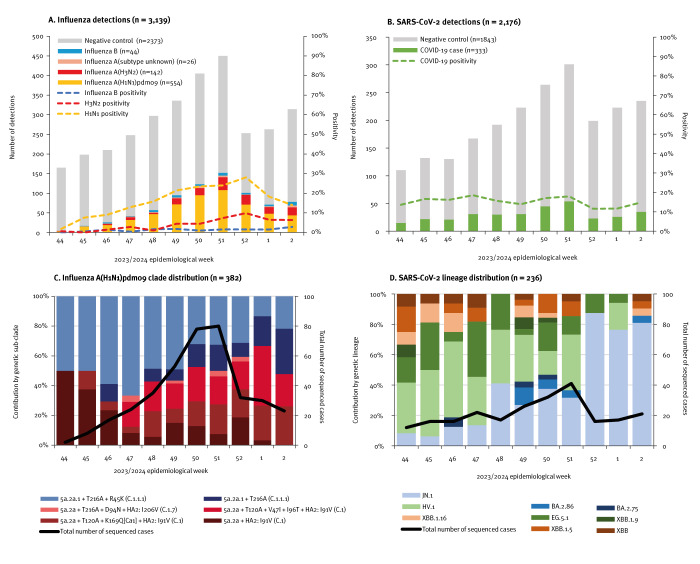
Influenza virus and SARS-CoV-2 test-positive and test-negative specimens, including genetic characterisation, by week of specimen collection, Canadian Sentinel Practitioner Surveillance Network, 29 October 2023–13 January 2024

Among 3,139 eligible specimens, 766 (24%) tested influenza-positive, including 722 (94%) influenza A and 44 (6%) influenza B (Figure 1). Of 696 (96%) subtyped influenza A viruses, 554 (80%) were A(H1N1)pdm09 and 142 (20%) were A(H3N2); we provide detailed influenza A virus HA sequencing findings in Supplementary Table S2. Of 554 A(H1N1)pdm09 viruses, 382 (69%) were HA-characterised and of these, 195 (51%) were vaccine-matched clade 5a.2a.1, whereas 187 (49%) were instead clade 5a.2a (Figure 1). Clade 5a.2a.1 contribution decreased over the study period, from 65% (33/51) of sequenced viruses in weeks 44–47 to 52% (127/246) in weeks 48–51 and 41% (35/85) in weeks 52–2 (p = 0.03). Across the analysis period approximately equal clade 5a.2a.1 vs 5a.2a contribution was observed in every province except BC, where 5a.2a.1 predominated overall (39/61; 64%). As appended in Supplementary Figure S1, clade 5a.2a.1 vs 5a.2a contribution by age group was also approximately equal except among participants 9-19 years for whom clade 5a.2a.1 appeared less frequent (16 of 42 sequenced viruses); age-related variation overall, however, was not significant (p = 0.49).

SARS-CoV-2: severe acute respiratory syndrome coronavirus 2.

Among eligible patients presenting with acute respiratory illness, the distribution of influenza virus A(H1N1)pdm09 genetic sub-clades and subgroups and SARS-CoV-2 genetic lineages among characterised case viruses are also displayed by week of specimen collection, with an overlayed weekly tally of sequenced case viruses. For influenza A(H1N1)pdm09, noted within a square bracket is an affected antigenic site, and in parentheses are corresponding NextStrain influenza subclade nomenclatures. Genetic characterisation details are appended for influenza A(H1N1)pdm09 in Supplementary Table S2 and for SARS-CoV-2 case viruses in Supplementary Table S3.

**Figure 2:**
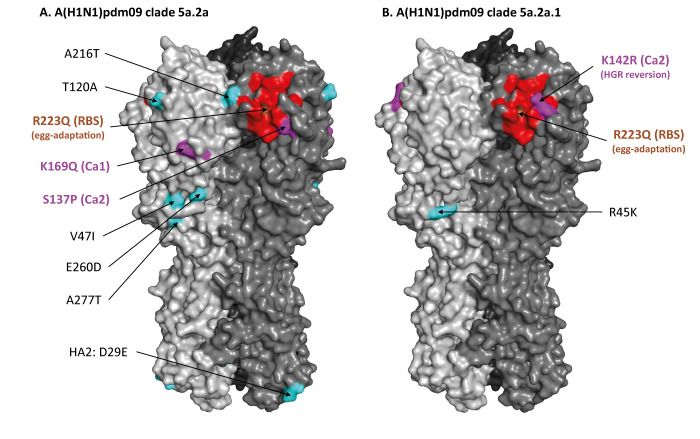
Haemagglutinin substitutions present in circulating influenza A(H1N1)pdm09 clade 5a.2a and 5a.2a.1 viruses detected by the Canadian Sentinel Practitioner Surveillance Network, relative to the egg-adapted high-growth reassortant vaccine strain IVR-238, 2023/24 influenza season

HGR: high-growth reassortant; RBS: receptor binding site.

Similar to the egg-adapted 5a.2a.1 vaccine strain and the associated high-growth reassortant (HGR) (called IVR-238), all SPSN 5a.2a.1 viruses bore the T216A substitution, most (144/195; 74%) with additional R45K. Conversely, few 5a.2a viruses (6/187; 3%) bore T216A, the remainder (181/187; 97%) instead had substitution I91V in the HA2 subunit. Among the latter, we identified two emergent T120A subgroups, one subgroup additionally including the K169Q(Ca1) substitution. Relative to the vaccine, circulating 5a.2a viruses also bore the S137P(Ca2) substitution. Both 5a.2a.1 and 5a.2a viruses further differed from the vaccine owing to a R223Q(RBS) mutation acquired by the vaccine virus during egg adaptation (Figure 2). Moreover, 5a.2a.1 (but not 5a.2a) viruses also differed from the vaccine due to a R142K(Ca2) reversion mutation in the HGR.

Representative influenza A(H1N1)pdm09 clade 5a.2a and 5a.2a.1 viruses were modelled in SWISS-MODEL [32] on A/Michigan/45/2015 (PDB 7knv.1) with substitutions highlighted in PyMOL relative to the egg-adapted HGR vaccine strain A/Victoria/4897/2022 IVR-238 [33]. The clade 5a.2a model shown in panel A depicts a combination of dominant T120A genetic clusters (Supplementary Table S2) rather than a single representative strain. The RBS is shown in red. Substitutions within the RBS are shaded brown. Substitutions in antigenic sites are shown in magenta and in cyan if non-antigenic. Amino acid numbering is based on A(H1)pdm09 numbering with the signal peptide removed. Vaccine-associated (vs circulating virus-associated) substitutions include Q223R, due to egg adaptation, and K142R, due to reversion in the HGR. Differences at position 216 may have an impact on glycosylation status as elaborated in the manuscript. Clade 5a.2a substitutions S137P (Ca2), E260D, A277T and HA2:D29E are clade-defining while other substitutions are new to one or more circulating 5a.2a subclades detected by the SPSN. The 5a.2a substitutions I96T (new) and HA2:H124N (clade-defining) are located inside the trimer interface and are therefore not visible in the structure.

Of the 142 influenza A(H3N2) viruses, 91 (64%) were HA-characterised. Most (88/91; 97%) were clade 2a.3a.1, of which most (77/88; 88%) additionally bore substitutions N122D(A)(−CHO) and K276E(C).

Among 2,176 specimens from participants in BC, Ontario and Quebec aged ≥ 12 years, 333 (15%) tested positive for SARS-CoV-2. Of 333 SARS-CoV-2 viruses, 236 (71%) were genetically characterised with lineage findings detailed in Supplementary Table S3. Numerous Omicron subvariants of interest were detected, including EG.5.1 (35/236; 15%), HV.1 (63/236; 27%) and JN.1 (90/236; 38%). Contribution by JN.1 increased across the analysis period from 11% (7/66) in weeks 44–47 to 34% (39/116) in weeks 48–51 and 81% (44/54) in weeks 52–2 (p < 0.001) (Figure 1).

## Epidemiological findings

Participant profiles are shown in Table 1 (influenza A) and Table 2 (COVID-19). The median age of influenza A cases was about 5 years younger than controls, whereas the median age of COVID-19 cases was about 5 years older than controls. Among ARI participants, > 80% in either analysis met ILI criteria. Among participants in COVID-19 analyses, 61% (1,053/1,738) self-reported a prior NAAT- or RAT-confirmed SARS-CoV-2 infection (excluding those for whom it was unknown) (Table 2), of whom 71% (750/1,053) reported that this had occurred ≥ 12 months prior to the current illness, 83% (871/1,053) ≥ 6 months prior and 88% (928/1,053) ≥ 3 months prior.

**Table 1:** Participant profile, influenza A analyses, Canadian Sentinel Practitioner Surveillance Network, 29 October 2023–13 January 2024 (n = 3,095)

Characteristics	All ARI participants (column %)							Influenza-vaccinated^a^ (row %, unless otherwise specified)						
	Overall		Influenza A cases		Influenza controls		p value^b^	Overall		p value^c^	Influenza A cases^d^		Influenza controls^d^	
	n	%	n	%	n	%		n	%		n	%	n	%
N (row %)	3,095	100	722	23	2,373	77	NA	823	27	NA	115	16	708	30
Age group (years)^e^														
1–8	546	18	150	21	396	17	< 0.001	85	16	< 0.001	13	9	72	18
9–19	317	10	80	11	237	10		48	15		11	14	37	16
20–49	1,151	37	293	41	858	36		217	19		36	12	181	21
50–64	568	18	125	17	443	19		166	29		27	22	139	31
65–79	407	13	65	9	342	14		226	56		22	34	204	60
≥ 80	106	3	9	1	97	4		81	76		6	67	75	77
Median (IQR)	39 (16–58)		35 (13–52)		40 (17–60)		< 0.001	57 (34–71)		< 0.001	47 (26–64)		58 (35–71)	
Sex														
Female	1,868	60	410	57	1,458	61	0.044	527	28	0.007	72	18	455	31
Male	1,208	39	303	42	905	38		288	24		41	14	247	27
Unknown	19	1	9	1	10	0	NA	8	42	NA	2	22	6	60
Comorbidity^f^														
No	2,055	66	520	72	1,535	65	< 0.001	428	21	< 0.001	66	13	362	24
Yes	693	22	129	18	564	24		294	42		37	29	257	46
Unknown	347	11	73	10	274	12	NA	101	29	NA	12	16	89	32
Meets ILI criteria														
No	136	4	16	2	120	5	< 0.001	29	21	0.202	1	6	28	23
Yes	2,483	80	636	88	1,847	78		652	26		104	16	548	30
Unknown	476	15	70	10	406	17	NA	142	30	NA	10	14	132	33
Province														
Alberta	369	12	120	17	249	10	< 0.001	98	27	< 0.001	23	19	75	30
BC	419	14	84	12	335	14		180	43		25	30	155	46
Ontario	1,502	49	371	51	1,131	48		386	26		58	16	328	29
Quebec	805	26	147	20	658	28		159	20		9	6	150	23
Specimen collection interval from onset of ARI^g^														
≤ 4 days	2,090	68	548	76	1,542	65	< 0.001	534	26	0.059	94	17	440	29
5–7 days	1,005	32	174	24	831	35		289	29		21	12	268	32
Weeks of specimen collection, 2023/24^g^														
44–47	816	26	84	12	732	31	< 0.001	142	17	< 0.001	11	13	131	18
48–51	1,466	47	405	56	1,061	45		409	28		68	17	341	32
52–2	813	26	233	32	580	24		272	33		36	15	236	41
XBB.1.5 vaccination (any, without regard to timing), restricted to BC, Ontario and Quebec only (all column %)^h^														
Total^i^	2,726	88	602	83	2,124	90	NA	725	88	NA	92	80	633	89
Yes	428	16	61	10	367	17	< 0.001	373	51	< 0.001	45	49	328	52
No	2,142	79	503	84	1,639	77		311	43		39	42	272	43
Unknown	156	6	38	6	118	6	NA	41	6	NA	8	9	33	5

ARI: acute respiratory illness; BC: British Columbia; ILI: Influenza like illness; IQR: interquartile range; NA: not applicable; SPSN: Canadian Sentinel Practitioner Surveillance Network.

^a^ Vaccination status based on participant or guardian report. Participants vaccinated < 2 weeks before onset of symptoms or with unknown vaccination status or timing were excluded.

^b^ p values for comparison between cases and controls were derived by two-way chi-squared test or Wilcoxon rank-sum test.

^c^ p values for comparison between vaccinated and not vaccinated were derived by two-way chi-squared test or Wilcoxon rank-sum test. The number not vaccinated can be derived by subtracting the number vaccinated from the total ARI participants, by row.

^d^ Without regard to time before illness onset, 131 of 738 (18%) cases and 814 of 2,479 (33%) controls across the analysis period were vaccinated (p < 0.001).

^e^ Children < 1 year excluded as per usual in prior SPSN analyses based on variability and/or uncertainty in their age-related vaccine eligibility over the course of the epidemic. Other age strata defined as per usual SPSN analyses predicated upon one- and two-dose recommendations for children ≥ 9 and < 9 years, respectively; higher likelihood of chronic comorbidity at ≥ 50 years; and higher age-associated risk among adults ≥ 65 years [31].

^f^ Includes chronic comorbidities that place individuals at higher risk of serious complications from influenza as defined by Canada’s National Advisory Committee on Immunization [31].

^g^ Missing specimen collection dates were imputed as the date the specimen was received and processed at the laboratory minus 2 days.

^h^ XBB.1.5 vaccination status based on provincial immunisation registry data from BC, Ontario and Quebec.

^i^ Total participants from BC, Ontario and Quebec, including those with unknown XBB.1.5 vaccination status used in the derivation of percentages displayed in all succeeding rows.

**Table 2:** Participant profile, COVID-19 analyses, participants ≥ 12 years in BC, Ontario and Quebec, Canadian Sentinel Practitioner Surveillance Network, 29 October 2023–13 January 2024 (n = 2,176)

Characteristics	All ARI participants (column %)							XBB.1.5-vaccinated^a^ (row %, unless otherwise specified)						
	Overall		COVID-19 cases		COVID-19 controls		p value^b^	Overall		p value^c^	COVID-19 cases^d^		COVID-19 controls^d^	
	n	%	n	%	n	%		n	%		n	%	n	%
N (row %)	2,176	100	333	15	1,843	85	NA	367	17	NA	40	12	327	18
Age group (years)^e^														
12–49	1,186	55	165	50	1,021	55	0.256	85	7	< 0.001	8	5	77	8
50–64	512	24	89	27	423	23		94	18		7	8	87	21
65–79	372	17	61	18	311	17		140	38		20	33	120	39
≥ 80	106	5	18	5	88	5		48	45		5	28	43	49
Median (IQR)	46 (32.5–63)		50 (36–64)		45 (32–62)		0.015	65 (52–74)		< 0.001	68 (57.5–76)		64 (51–74)	
Sex														
Female	1,402	64	208	62	1,194	65	0.420	234	17	0.826	22	11	212	18
Male	762	35	123	37	639	35		130	17		18	15	112	18
Unknown	12	1	2	1	10	1	NA	3	25	NA	0	0	3	30
Comorbidity^f^														
No	1,334	61	209	63	1,125	61	0.826	155	12	< 0.001	14	7	141	13
Yes	604	28	97	29	507	28		162	27		22	23	140	28
Unknown	238	11	27	8	211	11	NA	50	21	NA	4	15	46	22
Meets ILI criteria														
No	92	4	18	5	74	4	0.320	9	10	0.091	2	11	7	9
Yes	1,779	82	279	84	1,500	81		292	16		29	10	263	18
Unknown	305	14	36	11	269	15	NA	66	22	NA	9	25	57	21
Province														
British Columbia	308	14	20	6	288	16	< 0.001	94	31	< 0.001	5	25	89	31
Ontario	1,187	55	199	60	988	54		162	14		21	11	141	14
Quebec	681	31	114	34	567	31		111	16		14	12	97	17
Specimen collection interval from onset of ARI^g^														
≤ 4 days	1,397	64	246	74	1,151	62	< 0.001	222	16	0.104	32	13	190	17
5–7 days	779	36	87	26	692	38		145	19		8	9	137	20
Week of specimen collection, 2023/24^g^														
44–47	539	25	89	27	450	24	0.540	51	9	< 0.001	6	7	45	10
48–51	980	45	160	48	820	44		175	18		19	12	156	19
52–2	657	30	84	25	573	31		141	22		15	18	126	22
Self-reported prior NAAT- or RAT-confirmed SARS-CoV-2 infection^h^														
None	685	31	132	40	553	30	< 0.001	132	19	0.035	22	17	110	20
At least one	1,053	48	128	38	925	50		162	15		9	7	153	17
Unknown	438	20	73	22	365	20	NA	73	17	NA	9	12	64	18
Type of XBB.1.5 vaccine received (all column %)														
Spikevax ^i^	NA						NA	165	45	0.053^k^	15	38	150	46
Comirnaty ^j^								202	55		25	63	177	54
Nuvaxovid ^l^								0	0	NA	0	0	0	0
Number of COVID-19 vaccine doses in total, including XBB.1.5 (all column %)														
0–1	113	5	16	5	97	5	0.028	0	0	< 0.001^k^	0	0	0	0
2	558	26	79	24	479	26		3	1		0	0	3	1
3–5	1,272	58	205	62	1,067	58		170	46		11	28	159	49
6–7	233	11	33	10	200	11		194	53		29	73	165	50
Seasonal 2023/24 influenza vaccination (all column %)^m^														
Yes	636	29	91	27	545	30	0.447	320	87	< 0.001	39	98	281	86
No	1,448	67	226	68	1,222	66		38	10		0	0	38	12
Unknown	92	4	16	5	76	4	NA	9	2	NA	1	3	8	2

ARI: acute respiratory illness; BC: British Columbia; ILI: influenza like illness; IQR: interquartile range; NA: not applicable; NAAT: nucleic acid amplification; RAT: rapid antigen test; SARS-CoV-2: severe acute respiratory syndrome coronavirus 2; SPSN: Canadian Sentinel Practitioner Surveillance Network.

^a^ Vaccination status based on provincial immunisation registry data from BC, Ontario and Quebec. Participants vaccinated < 2 weeks before onset of symptoms or with unknown vaccination status or timing were excluded.

^b^ p values for comparison between cases and controls were derived by two-way chi-squared test or Wilcoxon rank-sum test.

^c^ p values for comparison between vaccinated and not vaccinated were derived by two-way chi-squared test or Wilcoxon rank-sum test, unless otherwise specified. The number not vaccinated can be derived by subtracting the number vaccinated from the total ARI participants, by row.

^d^ Without regard to timing before illness onset, 52 of 345 (15%) cases and 386 of 1,902 (20%) controls across the analysis period were vaccinated (p = 0.024).

^e^ Children < 12 years excluded. Other age strata defined as per usual SPSN influenza analyses based upon higher likelihood of chronic comorbidity at ≥ 50 years; and higher age-associated risk among adults ≥ 65 years, notably those ≥ 80 years.

^f^ Includes chronic comorbidities that place individuals at higher risk of serious complications from influenza as defined by Canada’s National Advisory Committee on Immunization [31].

^g^ Missing specimen collection dates were imputed as the date the specimen was received and processed at the laboratory minus 2 days.

^h^ Previously confirmed SARS-CoV-2 infection (confirmed by NAAT or RAT), as reported by participant or guardian. NAAT testing for SARS-CoV-2 was broadly available in Canada until early 2022 when self-administered RATs became widely accessible through private purchase and/or publicly funded access.

^i^ Andusomeran, monovalent mRNA COVID-19 (Spikevax XBB.1.5) vaccine manufactured by Moderna Biopharma Canada Corporation, authorised in Canada in September 2023. See: https://covid-vaccine.canada.ca/spikevax-xbb15/product-details

^j^ Raxtozinameran, monovalent mRNA COVID-19 (Pfizer-BioNTech Comirnaty Omicron XBB.1.5 subvariant) vaccine manufactured by BioNTech Manufacturing GmbH, authorised in Canada in September 2023. See: https://covid-vaccine.canada.ca/comirnaty-omicron-xbb15/product-details

^k^ p values were derived by one-way chi-squared test.

^l^ NVX-CoV2601, monovalent recombinant protein, adjuvanted COVID-19 (Nuvaxovid XBB.1.5 Omicron subvariant) vaccine manufactured by Novavax, authorised in Canada in December 2023. See: https://covid-vaccine.canada.ca/nuvaxovid-xbb15/product-details

^m^ Seasonal influenza vaccination status based on participant or guardian report (any, without regard to timing in relation to illness onset).

No exclusions based upon date of last non-XBB.1.5 vaccine dose have been applied to this table.

Among influenza A controls, 41% (236/580) were considered influenza-vaccinated during the latest epidemiological weeks 52–2, comparable to annual influenza vaccine coverage nationally (43% for the 2022/23 season among adults) [2]. Also comparable to national XBB.1.5 vaccine coverage estimates as of 3 December 2023 (15% among people ≥ 5 years) [14], 19% (30 of 162) of our SARS-CoV-2 controls in week 48 were considered vaccinated. Among participants considered influenza-vaccinated, a comparable proportion of cases and controls had also received the XBB.1.5 vaccine (45/84; 54% vs 328/600; 55%) (p = 0.85), whereas among participants considered XBB.1.5-vaccinated, all 39 of 39 SARS-CoV-2 cases vs 281 of 319 (88%) controls reported influenza vaccination (Fisher’s exact p = 0.02). Additional examination of the association between influenza and XBB.1.5 vaccine receipt and implications for VE estimation as per Doll et al. [6] are provided in Supplementary Table S4.

Adjusted influenza VE was 63% (95% CI: 51–72) against influenza A(H1N1)pdm09 and 40% (95% CI: 5–61) against A(H3N2) (Table 3). Influenza A(H1N1)pdm09 VE was lower against vaccine-matched clade 5a.2a.1 viruses at 56% (95% CI: 33–71) than alternate clade 5a.2a viruses at 67% (95% CI: 48–80). Influenza A(H1N1)pdm09 and A(H3N2) VE estimates were similar with restriction to ILI patients (61% and 46%, respectively); details of this subanalysis can be viewed in Supplementary Table S5. With age stratification, influenza A(H1N1)pdm09 VE estimates were higher in children and in older adults than younger adults, but with overlapping CI. 

**Table 3:** Vaccine effectiveness estimates against influenza A overall, by subtype and A(H1N1)pdm09 clade, Canadian Sentinel Practitioner Surveillance Network, 29 October 2023 (week 44)–13 January (week 2) 2024 (n = 3,095)

	Total	Cases			Controls			Unadjusted VE^a^		Adjusted VE^a,b^	
	N	n vac^c^	n	%	n vac^c^	n	%	%	95% CI	%	95% CI
Influenza A^d,e^	3,095	115	722	16	708	2,373	30	55	45–64	59	48–68
1–19 years^d^	863	24	230	10	109	633	17	44	10–65	60	34–76
20–64 years^d^	1,719	63	418	15	320	1,301	25	46	27–60	54	38–66
≥ 65 years ^d,f^	513	28	74	38	279	439	64	65	42–79	70	48–83
≥ 12 years, restricted to BC, Ontario, Quebec^e, g^	2,143	77	449	17	551	1,694	33	57	44–67	59	45–69
Influenza A(H1N1)pdm09^d,e^	2,927	81	554	15	708	2,373	30	60	48–69	63	51–72
1–19 years^d^	816	15	183	8	109	633	17	57	24–76	68	42–83
20–64 years^d^	1,617	46	316	15	320	1,301	25	48	27–63	56	38–69
≥ 65 years ^d,f^	494	20	55	36	279	439	64	67	41–82	72	47–85
≥ 12 years, restricted to BC, Ontario, Quebec^e, g^	2,020	52	326	16	551	1,694	33	61	46–71	63	48–74
Clade 5a.2a.1^d^	2,568	33	195	17	708	2,373	30	52	30–67	56	33–71
≥ 12 years, restricted to BC, Ontario, Quebec^e,g^	1,802	21	108	19	551	1,694	33	50	18–69	51	18–71
Clade 5a.2a^d^	2,560	23	187	12	708	2,373	30	67	49–79	67	48–80
≥ 12 years, restricted to BC, Ontario, Quebec^e,f,g^	1,799	12	105	11	551	1,694	33	73	51–85	73	48–86
Influenza A(H3N2)^d,e,f^	2,515	31	142	22	708	2,373	30	34	1–56	40	5–61
≥ 12 years, restricted to BC, Ontario, Quebec^e,f,g^	1,802	23	108	22	551	1,694	33	44	10–65	44	5–67

ARI: acute respiratory illness; BC: British Columbia; CI: confidence interval; ILI: influenza-like illness; OR: odds ratio; SARS-CoV-2: severe acute respiratory syndrome coronavirus 2; SPSN: Canadian Sentinel Practitioner Surveillance Network; vac: vaccinated; VE: vaccine effectiveness.

^a^ VE was calculated as 1 − OR × 100%. ORs compared test positivity between vaccinated and unvaccinated participants by logistic regression with covariate adjustment as specified. Unless otherwise specified, analyses include participants aged ≥ 1 year.

^b^ Adjusted for age group, province and calendar time, as specified.

^c^ Vaccination status based upon participant or guardian report. Participants vaccinated < 2 weeks before acute respiratory illness onset or with unknown vaccine status or timing were excluded.

^d^ Adjusted for age group (1–8, 9–19, 20–49, 50–64, 65–79, ≥ 80 years), province (Alberta, BC, Ontario, Quebec), and calendar time (single epidemiological weeks 44–2).

^e^ Sensitivity analyses are appended in Supplementary Table S5 and include additional adjustment for sex and comorbidity; analysis of VE against ILI (vs ARI) for comparison with prior SPSN estimates; and exclusion of SARS-CoV-2 test-positive cases from influenza test-negative controls to explore confounding by correlated influenza and COVID-19 vaccine status [7].

^f^ To address sparse data concerns, the use of Firth’s penalised logistic regression or collapsing of epidemiological weeks into biweekly categories for calendar time adjustment did not alter point estimates by more than 2%.

^g^ Restricted to participants aged ≥ 12 years enrolled in the provinces of BC, Ontario or Quebec for comparison with XBB.1.5 VE. Adjusted for age group (12–49, 50–64, 65–79, ≥ 80 years), province (BC, Ontario, Quebec), and calendar time single epidemiological weeks 44–2).

With restriction to participants ≥ 12 years in BC, Ontario and Quebec, influenza VE estimates were similar for influenza A(H1N1)pdm09 (63%), A(H3N2) (43%), and for A(H1N1)pdm09 clades 5a.2a.1 (51%) and 5a.2a (73%) (Table 3). In COVID-19 analyses with the same restrictions, additionally excluding recipients of non-XBB.1.5 vaccine ≤ 6 months before the launch of the autumn campaign, XBB.1.5 VE against COVID-19 was 47% (95% CI: 21–65) (Table 4). Age-stratified estimates of XBB.1.5 VE against COVID-19 are provided in Supplementary Table S7 and were comparable among those 12–64 and ≥ 65 years, also with broadly overlapping CI.

**Table 4:** XBB.1.5 vaccine effectiveness estimates against COVID-19 among participants ≥ 12 years in BC, Ontario and Quebec, Canadian Sentinel Practitioner Surveillance Network, 29 October 2023 (week 44)–13 January (week 2) 2024 (n = 2,176)

	Exclusion based upon timing of last non-XBB.1.5 vaccine dose											
	No exclusion for last dose				Receipt within 12 weeks prior^a^				Receipt within 24 weeks prior^b^			
	XBB.1.5 vaccinated^c^		Not XBB.1.5 vaccinated^c^	Total	XBB.1.5 vaccinated^c^		Not XBB.1.5 vaccinated^c^	Total	XBB.1.5 vaccinated^c^		Not XBB.1.5 vaccinated^c^	Total
	n	%	n	N	n	%	n	N	n	%	n	N
Total participants	367	17	1,809	2,176	366	17	1,799	2,165	342	16	1,770	2,112
Weeks since XBB.1.5 dose, median (IQR)	5 (3–8)		NA	NA	5 (3–8)		NA	NA	5 (3–8)		NA	NA
Weeks since last non-XBB.1.5 dose, median (IQR)	58 (51–64)		98 (67–115)	NA	58 (51–64)		98 (68–115)	NA	59 (53–65)		99 (70–115)	NA
Case participants	40	12	293	333	39	12	293	332	33	10	290	323
Weeks since XBB.1.5 dose, median (IQR)	5 (3–8)		NA	NA	5 (3–8)		NA	NA	5 (3–8)		NA	NA
Weeks since last non-XBB.1.5 dose, median (IQR)	58.5 (51–62)		97 (68–109)	NA	59 (51–62)		97 (68–109)	NA	61 (54–65)		97.5 (68–110)	NA
Control participants	327	18	1,516	1,843	327	18	1,506	1,833	309	17	1,480	1,789
Weeks since XBB.1.5 dose, median (IQR)	5 (3–8)		NA	NA	5 (3–8)		NA	NA	5 (3–8)		NA	NA
Weeks since last non-XBB.1.5 dose, median (IQR)	58 (51–65)		99 (66–115)	NA	58 (51–65)		99 (68–116)	NA	59 (53–65)		99 (71–116)	NA
Vaccine effectiveness	%		95% CI		%		95% CI		%		95% CI	
Unadjusted^d,e^	37		10–55		39		13–57		45		20–63	
Adjusted^e,f,g^	38		10–58		41		13–60		47		21–65	

BC: British Columbia; CI: confidence interval; IQR: interquartile range; NA: not applicable; OR: odds ratio.

^a^ Excludes participants who received a non-XBB.1.5 COVID-19 vaccine dose ≤ 12 weeks before provincial launch of the publicly funded XBB.1.5 vaccine campaign (i.e. receipt of non-XBB.1.5 vaccine on or after 19 July 2023 in BC, 7 August 2023 in Ontario and 18 July 2023 in Quebec).

^b^ Primary analysis: excludes participants who received a non-XBB.1.5 COVID-19 vaccine ≤ 24 weeks before provincial launch of the publicly funded XBB.1.5 vaccine campaign (i.e. receipt of non-XBB.1.5 vaccine on or after 26 April 2023 in BC, 15 May 2023 in Ontario and 25 April 2023 in Quebec).

^c^ Vaccination status based upon provincial immunisation registry data from BC, Ontario and Quebec. Participants vaccinated < 2 weeks before acute respiratory illness onset or with unknown vaccination status or timing were excluded.

^d^ VE was calculated as 1 − OR × 100%. ORs compared test positivity between vaccinated and unvaccinated participants by logistic regression with covariate adjustment as specified.

^e^ Sensitivity analyses are appended in Supplementary Table S6 and include additional adjustment for sex and comorbidity; exclusion of influenza test-positive cases from COVID-19 controls to explore confounding by correlated influenza and COVID-19 vaccine status [7]; and restriction to participants with previous SARS-CoV-2 infection confirmed by nucleic acid amplification test or rapid antigen test, as reported by participant or guardian.

^f^ Adjusted for age group (12–49, 50–64, 65–79, ≥ 80 years), province (BC, Ontario, Quebec), and calendar time (single epidemiological weeks 44–2).

Primary influenza A(H1N1)pdm09 and A(H3N2) VE estimates (63% and 40%, respectively) were relatively unaffected after exclusion of COVID-19 cases from influenza test-negative controls (64% and 38%, respectively). Excluding influenza cases from SARS-CoV-2 test-negative controls increased the primary XBB.1.5 VE estimate (47%) to 54% (95% CI: 31–70); the results of these additional analyses are appended in Supplementary Tables S5 and S6. With restriction to participants reporting prior NAAT- or RAT-confirmed SARS-CoV-2 infection, XBB.1.5 VE was higher at 67% (95% CI: 28–85) and when additionally excluding influenza cases from controls, it was 72% (95% CI: 39–87).

## Discussion

From late October 2023 through mid-January 2024, the Canadian SPSN estimates that the 2023/24 multivalent influenza vaccine reduced the risk of medically attended outpatient ARI due to influenza A(H1N1)pdm09 by about 60% and the risk due to influenza A(H3N2) by 40%. The updated autumn 2023 monovalent XBB.1.5 vaccine afforded a level of protection comparable to the seasonal influenza vaccine, reducing the risk of medically attended outpatient COVID-19 by about 50% overall. Among individuals reporting a prior confirmed SARS-CoV-2 infection, XBB.1.5 provided higher protection, reducing the COVID-19 risk by around 70% overall.

In mid-season analyses over the past decade, historical SPSN VE estimates against influenza A(H1N1)pdm09 have ranged approximately 45–75%, placing our current season’s estimates of 61–65% (including sensitivity analyses) at roughly mid-range [6], albeit higher than for the most recent prior A(H1N1)pdm09 epidemic in 2019/20 as reported by the SPSN (44%) [15], or other similar outpatient networks in the United States (US) (30%) [16] or Europe (48%) [17]. Our 2023/24 VE estimate is similar to the 61% reported earlier from Alberta, Canada for the shorter period spanning 29 October to 30 December 2023 [18]. In general, however, summary estimates mask important underlying complexity relevant to VE interpretation and comparison. To understand and improve vaccine performance against highly changeable viruses, molecular-level examination of the dynamic and evolving relationship between circulating and vaccine strains and their impact on protection is needed, such as routinely undertaken in integrated analyses each year by the SPSN.

We report paradoxical clade-specific variation in influenza A(H1N1)pdm09 VE, lower by 10% or more against vaccine-matched clade 5a.2a.1 than alternate clade 5a.2a viruses. While confidence intervals in VE comparisons overlapped and determinants of VE are multifactorial, unexpected mid-season findings inform hypotheses for further investigation. A predominant subset of clade 5a.2a.1 viruses, including vaccine and circulating strains, harbour the T216A substitution. This unique 5a.2a.1 substitution may have disrupted an important glycan within antigenic site Sa that has otherwise shielded all A(H1N1)pdm09 viruses from antibody binding since 2015 [19]. Selective loss of this protective glycan by 5a.2a.1 viruses (and the vaccine virus) may have influenced imprint-related effects, previously hypothesised to underpin age-specific variation in VE [20]. The Q223R substitution due to egg adaptation has affected every A(H1N1)pdm09 vaccine strain since 2009, but would not explain particular clade-specific variation in 2023/24 [21,22]. We instead flag another vaccine mutation uniquely accrued by the 2023/24 clade 5a.2a.1 vaccine HGR, namely R142K(Ca2) reversion. Present only in representative egg-derived HGRs of both inactivated and live attenuated influenza vaccines (but not their respective egg-adapted wildtype strains), this vaccine mutation may have resulted from selective pressure post-reassortment. Consequently, circulating 5a.2a.1 viruses are mismatched at R142, whereas clade 5a.2a viruses are naturally K142 matched to 2023/24 clade 5a.2a.1 vaccine HGRs. Notwithstanding higher VE against them, circulating clade 5a.2a viruses have other substitutions distinguishing them from the vaccine, including S137P(Ca2) and K169Q(Ca1). While both Ca2 residues 137 and 142 are proximal to the RBS, position 142 is in a more exposed area of the HA surface protein, and therefore potentially more influential on antibody response and VE.

To date in 2023/24, influenza A(H3N2) viruses have comprised just 20% of SPSN influenza A detections, most clade-mismatched to the vaccine and harbouring potential loss of glycosylation in antigenic site A. In that context, our VE estimates against influenza A(H3N2) ranging 38–46% (including sensitivity analyses) are lower than in 2022/23 (54%) [23], also lower than the Alberta estimate for 2023/24 (49%) [18], but comparable to historical mid-season SPSN estimates, typically < 50% [6]. 

The SPSN has no historical record against which to compare the autumn 2023 XBB.1.5 VE estimates, ranging 47–54% and higher at 67–72% among participants reporting prior SARS-CoV-2 infection, the latter now comprising the majority of the general population in Canada as elsewhere [24]. This observation is consistent with earlier studies indicating higher protection among people with hybrid (vaccine plus infection-induced) SARS-CoV-2 immunity [25]. Because testing for influenza virus, notably self-administered, has not been as broadly available, we could not conduct similar restriction for influenza VE estimation. A recent preprint of a US test-negative study among outpatients ≥ 18 years between 11 October and 10 December 2023 reported a similar XBB.1.5 VE estimate (58%) [26], while a screening study among patients ≥ 60 years in the Netherlands between 9 October and 5 December 2023 reported higher XBB.1.5 VE against COVID-19 hospitalisation (71%) [27]. To date, all mid-season 2023/24 analyses of XBB.1.5 (and influenza) VE have spanned just 1–2 months post-vaccination [18,26,27]. In addition, Omicron variants continue to diversify, with increasing contribution of BA.2.86 lineages, most notably JN.1. While BA.2.86 harbours more than 30 spike mutations relative to XBB.1.5, evidence so far suggests that immune evasion is not substantially greater than other variants [28,29]. Compared with its parent BA.2.86 lineage, however, JN.1 carries an additional mutation that enhances antigenic distinction and the capacity for antibody escape [28-30]. The SPSN mid-season XBB.1.5 VE estimate we report is in the context of JN.1 comprising < 40% of sequenced viruses overall. Increased circulation of the JN.1 variant across the analysis period, as also observed elsewhere, warrants further virological and VE monitoring.

We identified slight underestimation (< 10%) of COVID-19 VE associated with differences in the proportion of XBB.1.5-vaccinated COVID-19 cases vs controls who had also received the influenza vaccine, reflecting a potential bias due to an indirect confounding pathway [7]. Conversely, a more comparable proportion of vaccinated influenza cases and controls had reported XBB.1.5 vaccine receipt, with influenza VE estimates less affected. As explained in Supplementary Table S4, among people who came for XBB.1.5 vaccination, receipt of influenza vaccine appeared more likely than the reverse scenario of XBB.1.5 vaccine receipt among those coming for influenza vaccination. Our data suggest that confounding due to correlated receipt of vaccines against conditions included in the control series of test-negative designs may not be of the same magnitude for each vaccine considered. Rather, the bias will vary as the extent of correlated receipt of other vaccines varies for each vaccine’s separate VE analysis. These VE methodological considerations may become more relevant to explore as influenza, COVID-19 and soon respiratory syncytial virus vaccines (not yet widely available in Canada) are administered annually to the same target groups.

Our study is limited by small sample size and wide CIs, especially in stratified analyses, and by the potential for residual bias and confounding. Overlap in vaccine roll-out and epidemic evolution during mid-season analyses warrant further evaluation of calendar time effects through end-of-season analyses. We subjected as many as possible of our mid-season case viruses to WGS, resulting in the successful genetic characterisation of more than two-thirds of our influenza and COVID-19 case viruses. This supported clade-specific analysis and VE interpretation; however, we cannot rule out different patterns among uncharacterised case viruses or at the end of the season. In the absence of reliable influenza immunisation registries across participating provinces, we compared the proportion of test-negative controls for whom vaccine status was reported by the participant or sentinel practitioner with other population survey sources of vaccine coverage, shown to be reassuringly similar. Varied histories of prior infection (including imprinting) and vaccination may affect both influenza and COVID-19 VE in ways that require more in-depth immuno-epidemiological consideration. Our estimates are best interpreted in relation to the prevention of medically attended outpatient influenza or COVID-19. We cannot comment on VE against other more severe acute (e.g. hospitalisation) or long-term (e.g. long COVID) outcomes, although vaccines effective against infection are also expected to mitigate their associated downstream sequelae.

## Conclusions

In mid-season analysis, the 2023/24 influenza vaccine reduced the risk of medically attended outpatient ARI due to influenza A(H1N1)pdm09 by about 60%, paradoxically lower for vaccine-matched clade 5a.2a.1 than alternate clade 5a.2a viruses. Hypothesised contribution of mutations in circulating and vaccine strains warrants further biological, immunological and epidemiological investigation. Lower VE of 40% against influenza A(H3N2) is consistent with historical observations for that subtype. The updated autumn 2023 monovalent XBB.1.5 vaccine protected comparably well, reducing the risk of medically attended COVID-19 by about half overall, and by about two-thirds among previously infected individuals. Ongoing VE monitoring with increased time since vaccination and evolution in variant contribution is warranted.

## Supplementary Data

622000 Supplement
