# Supplementary material for: 2023/24 mid-season influenza and Omicron XBB.1.5 vaccine effectiveness estimates from the Canadian Sentinel Practitioner Surveillance Network (SPSN)
Source: Euro Surveill. 2024 Feb 15;29(7):2400076. doi: 10.2807/1560-7917.ES.2024.29.7.2400076 (PMC10986657; doi:10.2807/1560-7917.ES.2024.29.7.2400076)
Supplement: Supplement [file 24-00076_SKOWRONSKI_Supplement.pdf]

Supplementary Materials

This supplementary material is hosted by *Eurosurveillance* as supporting information alongside the article “*2023/24 midseason influenza and Omicron XBB.1.5 vaccine effectiveness estimates from the Canadian Sentinel Practitioner Surveillance Network (SPSN)*” on behalf of the authors who remain responsible for the accuracy and appropriateness of the content. The same standards for ethics, copyright, attributions and permissions as for the article apply. Supplements are not edited by *Eurosurveillance* and the journal is not responsible for the maintenance of any links or email addresses provided therein.

Table of Contents (Hyperlinked)

Supplementary Table S1. Details related to hemagglutinin (HA) sequences of original, cell-passaged, egg-passaged and high growth reassortant (HGR) versions of the 2023/24 inactivated (A/Victoria/4897/2022) and live attenuated (A/Norway/31694/2022) influenza vaccine strains of A(H1N1)pdm09 3

Supplementary Table S2. Influenza A case viruses (n=382) by genetic subgroup in vaccine effectiveness analyses, Canadian Sentinel Practitioner Surveillance Network (SPSN), 29 October (week 44) 2023 – 13 January (week 2) 2024 4

Supplementary Table S3. SARS-CoV-2 case viruses (n=236) by genomic lineage in vaccine effectiveness analyses, Canadian Sentinel Practitioner Surveillance Network (SPSN), 29 October (week 44) 2023 – 13 January (week 2) 2024 5

Supplementary Figure S1. Distribution of influenza A(H1N1)pdm09 clade 5a.2a.1 (n=195) and clade 5a.2a (n=187) case viruses by age group, Canadian Sentinel Practitioner Surveillance Network (SPSN), 29 October (week 44) 2023 – 13 January (week 2) 2024 6

Supplementary Table S4. Examination of potential bias due to correlated influenza and XBB.1.5 vaccine receipt 7

Supplementary Table S5. Sensitivity influenza vaccine effectiveness estimates against influenza A overall, by subtype and A(H1N1)pdm09 clade, Canadian Sentinel Practitioner Surveillance Network (SPSN), 29 October (week 44) 2023 – 13 January (week 2) 2024 8

Supplementary Table S6. Sensitivity XBB.1.5 vaccine effectiveness estimates against COVID-19 among participants ≥ 12 years in BC, Ontario and Quebec, Canadian Sentinel Practitioner Surveillance Network (SPSN), 29 October (week 44) 2023 – 13 January (week 2) 2024 10

Supplementary Table S7. Age-stratified XBB.1.5 vaccine effectiveness estimates against COVID-19 among participants 12-64 years and ≥ 65 years in BC, Ontario and Quebec, Canadian Sentinel Practitioner Surveillance Network (SPSN), 29 October (week 44) 2023 – 13 January (week 2) 2024 12

References, Supplementary Material 13

Supplementary Table S1. Details related to hemagglutinin (HA) sequences of original, cell-passaged, egg-passaged and high growth reassortant (HGR) versions of the 2023/24 inactivated (A/Victoria/4897/2022) and live attenuated (A/Norway/31694/2022) influenza vaccine strains of A(H1N1)pdm09

Sequences were downloaded from the Global Initiative on Sharing All Influenza Data (GISAID) with details displayed below [1].

Segment ID	Country	Collection date	Isolate-ID	Isolate name	Originating Lab	Submitting Lab	Authors
EPI2320530	Australia	2022-Oct-02	EPI_ISL_16733796	A/Victoria/4897/2022	Royal Melbourne Hospital	WHO Collaborating Centre for Reference and Research on Influenza	Soppe, S; Rynehart, C; Peck, H; Aziz, A
EPI2235969	Australia	2022-Oct-02	EPI_ISL_16003490	A/Victoria/4897/2022			Deng, Y-M; Barr, I; Aziz,A
EPI2319193	Australia	2022-Oct-02	EPI_ISL_16714268	A/Victoria/4897/2022			
EPI2397519	Australia	2022-Oct-02	EPI_ISL_16899235	A/Victoria/4897/2022			
EPI2439437	Australia	2023-Jan-01	EPI_ISL_17086692	IVR-238 (A/Victoria/4897/2022)	Seqirus Pty Ltd (CSL Group)		
EPI2437457	Australia	2022-Oct-02	EPI_ISL_17072386	A/Victoria/4897/2022 (22/316)	WHO Collaborating Centre for Reference and Research on Influenza	National Institute for Biological Standards and Control (NIBSC)	Nicolson, Carolyn
EPI2437459	Australia	2022-Oct-02	EPI_ISL_17072387	A/Victoria/4897/2022 IVR-238 (22/318)	Seqirus Pty Ltd (CSL Group)		
EPI2805564	Australia	2022-Oct-02	EPI_ISL_18557516	A/Victoria/4897/2022 IVR-238 (23/250)	Victoria Infectious Diseases Reference Laboratory		
EPI2805566	Australia	2022-Oct-02	EPI_ISL_18557517	A/Victoria/4897/2022 (23/252)			
EPI2461771	Australia	2022-Oct-02	EPI_ISL_17206439	IVR-238 A/Victoria/4897/2022 (20230302A)	Seqirus Pty Ltd (CSL Group)	WHO Chinese National Influenza Center	
EPI2603720	Australia	2022-Oct-02	EPI_ISL_17830834	A/Victoria/4897/2022	WHO Collaborating Centre for Reference and Research on Influenza	Centers for Disease Control and Prevention	
EPI2447516	Australia	2022-Oct-02	EPI_ISL_17102775	A/Victoria/4897/2022			
EPI2213676	Norway	2022-Sep-24	EPI_ISL_15728546	A/Norway/31694/2022	Norwegian Institute of Public Health	Norwegian Institute of Public Health	Bragstad, K; Hungnes, O; Madsen, M, P; Rohringer, A; Riis, R
EPI2603712	Norway	2022-Sep-24	EPI_ISL_17830833	A/Norway/31694/2022	National Institute for Medical Research	Centers for Disease Control and Prevention	
EPI2238868	Norway	2022-Sep-24	EPI_ISL_16043978	A/Norway/31694/2022	WHO National Influenza Centre	Crick Worldwide Influenza Centre	
EPI2495553	Norway	2022-Sep-24	EPI_ISL_17352907	A/Norway/31694/2022 (22/312)	Crick Worldwide Influenza Centre	National Institute for Biological Standards and Control (NIBSC)	Nicolson, Carolyn
EPI2495559	Norway	2022-Sep-24	EPI_ISL_17352969	A/Norway/31694/2022 NIB-133 (23/112)			

Supplementary Table S2. Influenza A case viruses (n=382) by genetic subgroup in vaccine effectiveness analyses, Canadian Sentinel Practitioner Surveillance Network (SPSN), 29 October (week 44) 2023 – 13 January (week 2) 2024

**Genetic clade, as defined by ECDC [2] based upon specified amino acid substitutions**[1,2]<br>+ additional substitutions identified (antigenic site)	**NextStrain subclade [3]**	**BC**<br>n (%)	**Alberta**<br>n (%)	**Ontario**<br>n (%)	**Québec**<br>n (%)	**TOTAL**<br>n (%)
**Influenza A(H1N1)pdm09,** N (case viruses)<br>Case viruses sequenced, n (% n/N)		**68**<br>**61 (90%)**	**105**<br>**81 (77%)**	**244**<br>**143 (59%)**	**137**<br>**97 (71%)**	**554**<br>**382 (69%)**
**5a.2a = 5a.2 + K54Q + A186T (Sb) + Q189E (Sb) + E224A (RBS) + R259K + K308R**	C.1	**22 (36%)**	**40 (49%)**	**71 (50%)**	**54 (56%)**	**187 (49%)**
+ HA2: I91V[3]		2	6	19	19	43
+ T120A + K169Q (Ca1) + HA2: I91V		2	7	24	29	62
+ T120A + V47I + I96T + HA2: I91V		17	27	24	5	76
+ T216A + D94N + HA2: I206V	C.1.7	1		4	1	6
**5a.2a.1 = 5a.2a + P137S (Ca2) + K142R (Ca2) + D260E + T277A + HA2: E29D + I91V + N124H**	C.1.1	**39 (64%)**	**41 (51%)**	**72 (50%)**	**43 (44%)**	**195 (51%)**
+ T216A[4,5]	C.1.1.1	9	4	24	14	51
+ T216A[5] + R45K		30	37	48	29	144
**Influenza A(H3N2),** N (case viruses)<br>Case viruses sequenced, n (% n/N)		**12**<br>**9 (75%)**	**5**<br>**4 (80%)**	**122**<br>**76 (63%)**	**3**<br>**2 (67%)**	**142**<br>**91 (64%)**
**2a.1b = 2a + D53G (C) + D104G + K276R (C) + I140K (A) + R299K (C)**	G.1.1.2			**2 (3%)**		**2 (%)**
**2a.3a = 2a + D53N (C) + N96S (D)(+CHO) + I192F (B) + E50K (C) + HA2: N49S**	G.1.3.1			**1 (1%)**		**1 (%)**
**2a.3a.1 = 2a.3a + I140K (A) + I223V**	H	**9 (100%)**	**4 (100%)**	**73 (96%)**	**2 (100%)**	**88 (97%)**
+ I25V + HA2: V18M + I89V	H.1	1	1	3		5
+ N122D (A)(-CHO) + K276E (C)[6]	H.2	7	2	67	1	77
+ K276E (C) + Q173R (D)	H.4		1		1	2

BC = British Columbia; ECDC = European Centre for Disease Control and Prevention; -CHO = loss of potential glycosylation site; RBS = receptor binding site. A(H1N1)pdm09 clade colour coding aligns with **Figure 1 (panel C)** of the main manuscript.

[1] Influenza A(H1N1)pdm09 substitutions relative to A/Wisconsin/588/2019 5a.2 reference virus and Influenza A(H3N2) substitutions relative to A/Darwin/6/2021 2a reference virus.
[2] WGS performed using Oxford Nanopore technology with inclusion criteria of ≥50X depth of coverage across ≥90% of the HA segment; Sanger sequencing used to supplement insufficient HA sequence data.
[3] Includes 13 viruses with additional substitutions D269N, P137S (Ca2)
[4] Includes 24 viruses with additional substitutions R113K, HA2: V100I.
[5] Potential glycosylation impacts; see discussion for explanation.
[6] Includes 20 viruses with reversion E276K (C)

Supplementary Table S3. SARS-CoV-2 case viruses (n=236) by genomic lineage in vaccine effectiveness analyses, Canadian Sentinel Practitioner Surveillance Network (SPSN), 29 October (week 44) 2023 – 13 January (week 2) 2024

Whole genome sequencing of SPSN SARS-CoV-2 case viruses followed routine provincial or national laboratory protocols [4-12], and contemporary Pango nomenclature [13,14]. See **Supplementary Material references** for details.

Genetic lineage, as defined by Pango nomenclature: Parental lineage[1]	Sub-lineages detected in SPSN samples[2,3]	British Columbia	Ontario	Québec	TOTAL
**Case viruses, N**		**20**	**199**	**114**	**333**
Case viruses sequenced, n (% n/N)		**14 (70%)**	**125 (63%)**	**97 (85%)**	**236 (71%)**
XBB	XCH.*, FY.5, XCP, GW.5, GS.4.1		6	3	9
XBB.1.5	JD.1.*, GK.1.*, HR.1, HY.1		12	3	15
XBB.1.16	JF.1	3	2	4	9
XBB.1.9	FL.1.5.*		2	2	4
EG.5.1	JG.3, HK.*	1	22	12	35
HV.1		3	34	26	63
BA.2.75	DV.7.1.*		1	2	3
BA.2.86	JN.*		6	2	8
JN.1		7	40	43	90

Clade colour coding aligns with **Figure 1 (panel D)** of the main manuscript.

[1] All lineages include their descendants unless otherwise listed.
[2] Only lineages with unique Pango designations noted here.
[3] Asterisks indicates the lineage includes any descendants, except ones displayed separately.

Supplementary Figure S1. Distribution of influenza A(H1N1)pdm09 clade 5a.2a.1 (n=195) and clade 5a.2a (n=187) case viruses by age group, Canadian Sentinel Practitioner Surveillance Network (SPSN), 29 October (week 44) 2023 – 13 January (week 2) 2024

The percentage of sequenced A(H1N1)pdm09 viruses that were clade 5a.2a.1 versus clade 5a.2a sequenced by age group are displayed below, with the tally (n) of clade-specific viruses overlayed.

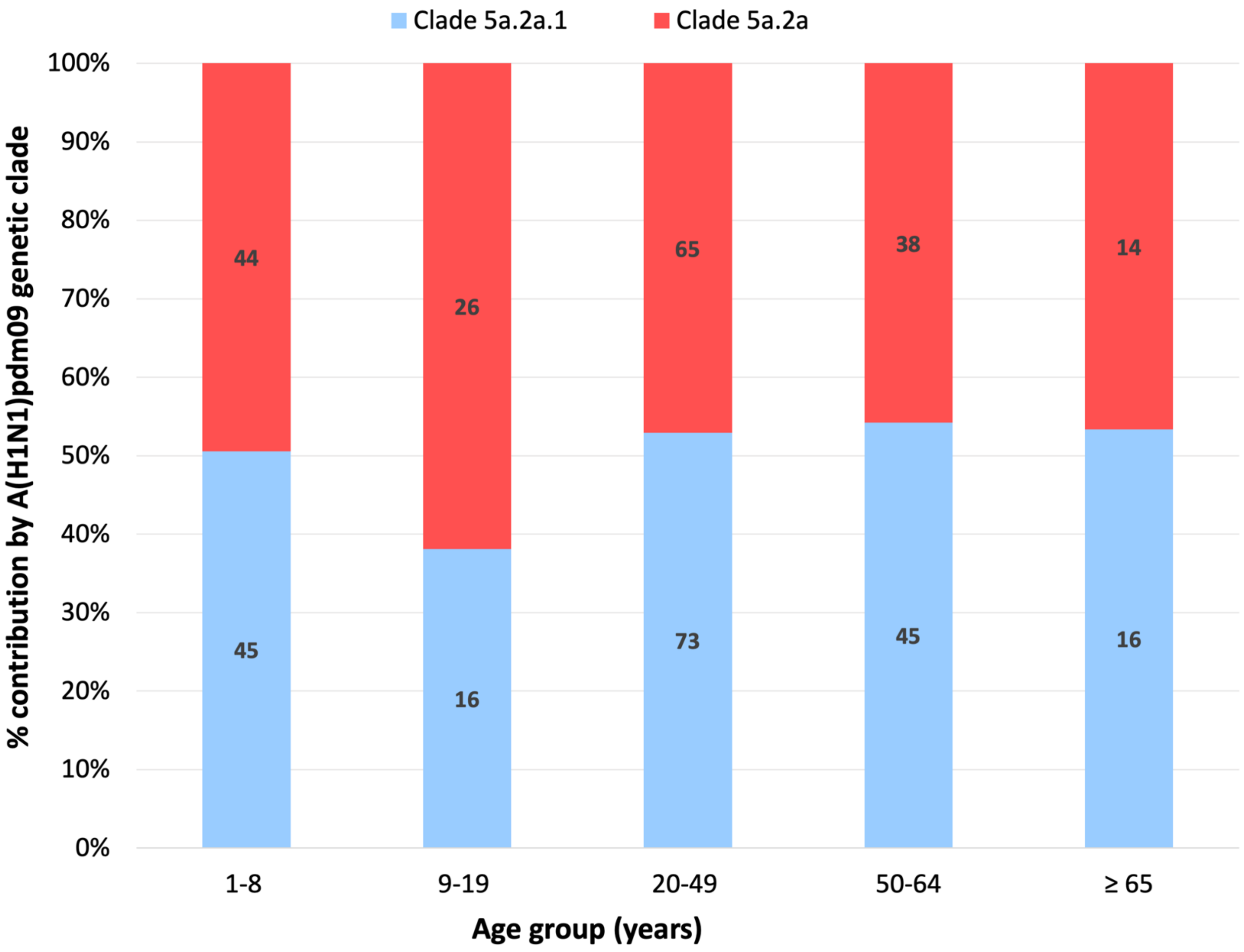

Supplementary Table S4. Examination of potential bias due to correlated influenza and XBB.1.5 vaccine receipt

A core principle for valid vaccine effectiveness (VE) estimation by the test-negative case-control design (TND) is that the evaluated vaccine must have no effect upon other aetiologies (e.g., viruses) causing the same disease (e.g., acute respiratory illness) included in the test-negative control series [15]. For vaccines, this principle is generally respected owing to the highly specific nature of vaccine-induced protection toward the target pathogen of interest, with no effect on non-target pathogens and as validated for influenza vaccine in TND analysis of randomized controlled datasets [15].

However, Doll et al have raised a potential bias due to correlated receipt of other vaccines [16]. Such bias does not require a direct causal or biological association between the vaccine of interest and non-target pathogens included in the control series and does not represent a violation of the core TND principle. Rather it reflects the influence of correlated vaccines received and *their* impacts on other aetiologies within the control series (i.e., an indirect confounding pathway). Within our mid-season SPSN dataset, we explored this potential bias through crude examination of participant profiles (restricted to provinces of British Columbia (BC), Ontario and Quebec providing XBB.1.5 vaccine information), as shown unadjusted in the table below, and in VE sensitivity analyses with covariate adjustment, as shown in **Supplementary Tables S5 and S6**.

As expected for protective virus-specific vaccines, across the analysis period, the percentage considered influenza vaccinated within our dataset was lower among influenza A cases than controls (84/564; 15% versus 600/2006; 30% (p<0.001). Similarly, the percentage considered XBB.1.5 vaccinated was lower among COVID-19 cases than controls (39/317; 11% versus 319/1767; 18%) (p=0.01).

Also consistent with virus-specific vaccines, among participants considered not vaccinated against influenza, the percentage who were vaccinated with XBB.1.5 vaccine did not differ among influenza A cases versus controls (16/480; 3% versus 39/1406; 3%) (p=0.53). Similarly, among participants who were not vaccinated with XBB.1.5 vaccine, the percentage who were vaccinated with influenza vaccine did not differ between COVID-19 cases versus controls (52/278; 19% versus 264/1448; 18%) (p=0.85).

Among participants considered influenza vaccinated, a comparable proportion of influenza A cases and controls had also received the XBB.1.5 vaccine (45/84; 54% versus 328/600; 55%) (p=0.85). Conversely, among participants considered XBB.1.5 vaccinated, all (39/39) COVID-19 cases versus 281/319 (88%) controls reported influenza vaccination (Fisher exact p=0.02).

Influenza A analyses (Restricted to BC, Ontario and Quebec and ≥ 1 year)	Influenza A Cases			Influenza Test-negative Controls			Total
	Influenza vaccinated[1]	Not influenza vaccinated	Total	Influenza vaccinated[1]	Not influenza vaccinated	Total	
XBB.1.5 vaccinated[2]	45 (54%)[3]	16 (3%)[4]	61	328 (55%)[3]	39 (3%)[4]	367	428
Not XBB.1.5 vaccinated	39	464	503	272	1367	1639	2142
Total	84 (15%)[5]	480	564	600 (30%)[5]	1406	2006	2570
**COVID-19 analyses[6] (Restricted to BC, Ontario and Quebec and ≥ 12 years)**	**COVID-19 Cases**			**SARS-CoV-2 Test-negative Controls**			**Total**
	XBB.1.5 Vaccinated[1]	Not XBB.1.5 Vaccinated	Total	XBB.1.5 Vaccinated[1]	Not XBB.1.5 Vaccinated	Total	
Influenza vaccinated[2]	39 (100%)[7]	52 (19%)[8]	91	281 (88%)[7]	264 (18%)[8]	545	636
Not influenza vaccinated	0	226	226	38	1184	1222	1448
Total	39 (11%)[9]	278	317	319 (18%)[9]	1448	1767	2084

Among controls included in COVID-19 analyses, nearly 90% who received XBB.1.5 vaccine also received influenza vaccine; whereas, among influenza controls, just slightly more than half who received influenza vaccine also received XBB.1.5 vaccine. Based upon the above, it appears that among those who came to receive XBB.1.5 vaccine, co-administration of influenza vaccine was more likely than the reverse scenario of XBB.1.5 vaccine co-administration among those coming for influenza vaccine.

In our dataset, the inclusion of influenza cases in the COVID-19 control series could lead to under-estimation of XBB.1.5 VE. This is because with protective influenza vaccine, influenza cases are less likely to be influenza vaccinated and given associated XBB.1.5 vaccine receipt, also less likely to be XBB.1.5 vaccinated. To assess this signal of possible bias in adjusted VE analyses, we removed influenza cases (both influenza A and B) from the COVID-19 control series and also explored removing COVID-19 cases from the influenza control series, as shown in **Supplementary Tables S5 and S6**.

[1] Vaccinated ≥ 2 weeks prior to onset of acute respiratory illness. Participants vaccinated < 2 weeks before onset or with unknown vaccination status/timing excluded.
[2] Any receipt of the specified vaccine without regard to timing in relation to illness onset (unknown vaccination status excluded).
[3] Among participants considered influenza vaccinated, comparison of percent XBB.1.5 vaccinated between influenza cases versus controls, chi-square p = 0.85
[4] Among participants considered not influenza vaccinated, comparison of percent XBB.1.5 vaccinated between influenza cases versus controls, chi-square p = 0.53
[5] Among influenza A cases versus controls, comparison of percent considered influenza vaccinated, chi-square p <0.001.
[6] As per Table 2 of the main manuscript, no exclusions based upon date of the last non-XBB.1.5 vaccine dose are applied in this exploration.
[7] Among participants considered XBB.1.5 vaccinated, comparison of percent influenza vaccinated between COVID-19 cases versus controls, Fisher's exact p = 0.019
[8] Among participants considered not XBB.1.5 vaccinated, comparison of percent influenza vaccinated between COVID-19 cases versus controls, chi-square p = 0.85
[9] Among COVID-19 cases versus controls, comparison of percent considered XBB.1.5 vaccinated by chi-square, p =0.01.

Supplementary Table S5. Sensitivity influenza vaccine effectiveness estimates against influenza A overall, by subtype and A(H1N1)pdm09 clade, Canadian Sentinel Practitioner Surveillance Network (SPSN), 29 October (week 44) 2023 – 13 January (week 2) 2024

	Total	Cases		Controls		Unadjusted VE[a]		Adjusted VE[a,b]	
	N	n vac[c]/N	%	n vac[c]/N	%	%	95% CI	%	95% CI
**With additional sex and comorbidity adjustment[d]**									
Influenza A[e]	2729	101/640	16	613/2089	29	55	43–64	58	46–68
1-19 years[e]	757	21/197	11	99/560	18	44	8–66	63	35–79
20-64 years[e]	1514	54/375	14	270/1139	24	46	26–61	53	35–67
≥ 65 years [e,f]	458	26/68	38	244/390	63	63	37–78	68	43–83
≥ 12 years, restricted to BC, Ontario, Quebec[g]	1906	68/409	17	472/1497	32	57	43–67	57	42–69
Influenza A(H1N1)pdm09[e]	2579	71/490	14	613/2089	29	59	47–69	62	49–72
1-19 years[e]	715	13/155	8	99/560	18	57	22–77	70	42–84
20-64 years[e]	1424	40/285	14	270/1139	24	47	25–63	56	35–70
≥ 65 years [e,f]	440	18/50	36	244/390	63	66	38–82	71	44–85
≥ 12 years, restricted to BC, Ontario, Quebec[g]	1796	46/299	15	472/1497	32	61	45–72	62	45–73
5a.2a.1[e]	2262	29/173	17	613/2089	29	52	27–68	55	30–71
≥ 12 years, restricted to BC, Ontario, Quebec[g]	1597	19/100	19	472/1497	32	49	15–69	49	11–70
5a.2a[e]	2256	21/167	13	613/2089	29	65	45–78	67	46–80
≥ 12 years, restricted to BC, Ontario, Quebec[f,g]	1593	10/96	10	472/1497	32	75	51–87	75	49–88
Influenza A(H3N2)[e,f]	2214	27/125	22	613/2089	29	34	-3 to 57	41	4–63
≥ 12 years, restricted to BC, Ontario, Quebec[f,g]	1592	20/95	21	472/1497	32	42	4–65	44	0–68
**Excluding COVID-19 cases from influenza test-negative controls**									
Influenza A[h]	2694	115/722	16	591/1972	30	56	45–65	60	49–69
1-19 years[h]	823	24/230	10	99/593	17	42	7–64	57	29–75
20-64 years[h]	1445	63/418	15	264/1027	26	49	31–62	57	41–69
≥ 65 years [f,h]	426	28/74	38	228/352	65	67	44–80	72	50–84
≥ 12 years, restricted to BC, Ontario, Quebec[i]	1807	77/449	17	457/1358	34	59	47–69	61	48–71
Influenza A(H1N1)pdm09[h]	2526	81/552	15	591/1972	30	60	48–69	64	53–73
1-19 years[h]	776	15/183	8	99/593	17	55	21–75	66	37–81
20-64 years[h]	1343	46/316	15	264/1027	26	51	31–65	60	42–72
≥ 65 years [f,h]	407	20/55	36	228/352	65	69	44–83	73	49–86
≥ 12 years, restricted to BC, Ontario, Quebec[i]	1684	52/326	16	457/1358	34	63	49–73	65	51–75
5a.2a.1[h]	2167	33/195	17	591/1972	30	52	30–68	58	36–72
≥ 12 years, restricted to BC, Ontario, Quebec[i]	1466	21/108	19	457/1358	34	52	22–71	55	24–74
5a.2a[h]	2159	23/187	12	591/1972	30	67	49–79	68	49–80
≥ 12 years, restricted to BC, Ontario, Quebec [f,i]	1463	12/105	11	457/1358	34	75	53–86	74	51–87
Influenza A(H3N2) [f,h]	2114	31/142	22	591/1972	30	35	2–57	38	3–61
≥ 12 years, restricted to BC, Ontario, Quebec [f,i]	1466	23/108	21	457/1358	34	47	14–67	44	5–67
**Restricted to participants with influenza-like illness (ILI)[j]**									
Influenza A[k]	2483	104/636	16	548/1847	30	54	42–63	58	45–67
Influenza A(H1N1)pdm09[k]	2335	75/488	15	548/1847	30	57	44–67	61	47–71
Influenza A(H3N2) [f,k]	1974	26/127	20	548/1847	30	39	5–61	46	11–67

BC: British Columbia; VE: vaccine effectiveness; CI: confidence interval; vac = vaccinated

[a] VE was calculated as 1 − odds ratios (OR) × 100%. ORs compared test positivity between vaccinated and unvaccinated participants by logistic regression with covariate adjustment as specified. Unless otherwise specified, analyses include participants aged ≥ 1 years.
[b] All VE analyses adjusted for age group, province and calendar time as specified
[c] Vaccination status based upon participant or guardian report. Participants vaccinated < 2 weeks before acute respiratory illness onset or with unknown vaccine status or timing were excluded.
[d] Unlike the primary analysis, excludes individuals missing sex and comorbidity information and additionally adjusts for sex and comorbidity.
[e] Adjusted for age group (1–8, 9–19, 20–49, 50–64, 65-79, ≥ 80 years), province (Alberta, BC, Ontario, Quebec), calendar time (single epi-weeks 44-2), and additionally sex (male, female) and comorbidity (yes, no).
[f] To address sparse data concerns, the use of Firth's penalized logistic regression or collapsing of epi-weeks into biweekly categories for calendar time adjustment did not alter point estimates by more than 2% (absolute) [not displayed].
[g] Restricted to participants aged ≥ 12 years enrolled in BC, Ontario or Quebec for comparison with XBB.1.5 VE. Adjusted for age group

(12-49, 50-64, 65-79, ≥ 80 years), province (BC, Ontario, Quebec), calendar time (single epi-weeks 44-2), and additionally sex (male, female) and comorbidity (yes, no).

[h] Adjusted for age group (1–8, 9–19, 20–49, 50–64, 65-79, ≥ 80 years), province (Alberta, BC, Ontario, Quebec), and calendar time (single epi-weeks 44-2). Additional adjustment for sex and comorbidity (excluding participants with unknown data) did not alter any point estimates by more than 4% (absolute) [not displayed].

[i] Restricted to participants aged ≥ 12 years enrolled in BC, Ontario or Quebec for comparison with XBB.1.5 VE. Adjusted for age group (12-49, 50-64, 65-79, ≥ 80 years), province (BC, Ontario, Quebec), and calendar time (single epi-weeks 44-2). Additional adjustment for sex and comorbidity (excluding participants with unknown data) did not alter point estimates by more than 3% (absolute) [not displayed].

[j] Restricted to participants with ILI for comparison with VE estimates from prior seasons. ILI defined as per historic SPSN analyses as fever plus cough plus one or more of sore through, myalgia, arthralgia, or prostration. Fever is not required for elderly adults ≥ 65 years.

[k] Adjusted for age group (1–8, 9–19, 20–49, 50–64, 65-79, ≥ 80 years), province (Alberta, BC, Ontario, Quebec), and calendar time (single epi-weeks 44-2). Additional adjustment for sex and comorbidity (excluding participants with unknown data) did not alter point estimates by more than 2% (absolute) [not displayed].

Supplementary Table S6. Sensitivity XBB.1.5 vaccine effectiveness estimates against COVID-19 among participants ≥ 12 years in BC, Ontario and Quebec, Canadian Sentinel Practitioner Surveillance Network (SPSN), 29 October (week 44) 2023 – 13 January (week 2) 2024

**With additional sex and comorbidity adjustment[a,b]**	**XBB.1.5 vaccinated[c]**		**Not XBB.1.5 vaccinated[c]**	**Total**
	**n**	**%**	**n**	**N**
Total participants	295	16	1576	1871
Weeks since XBB.1.5 dose, median (IQR)	5 (3-8)		NA	NA
Weeks since last non-XBB.1.5 dose, median (IQR)	59 (53-65)		99 (70-115)	NA
Case participants	31	10	266	297
Weeks since XBB.1.5 dose, median (IQR)	5 (3-8)		NA	NA
Weeks since last non-XBB.1.5 dose, median (IQR)	61 (54-65)		97 (68-109)	NA
Control participants	264	17	1310	1574
Weeks since XBB.1.5 dose, median (IQR)	6 (3-8)		NA	NA
Weeks since last non-XBB.1.5 dose, median (IQR)	59 (53-65)		99 (70-116)	NA
Vaccine effectiveness	%		95% CI	
Unadjusted[d]	42		14–61	
Adjusted[d,e]	44		14–63	
**Excluding influenza test-positive cases from SARS-CoV-2 test-negative controls[b]**	**XBB.1.5 vaccinated[c]**		**Not XBB.1.5 vaccinated[c]**	**Total**
	**n**	**%**	**n**	**N**
Total participants	289	17	1371	1660
Weeks since XBB.1.5 dose, median (IQR)	5 (3-8)		NA	NA
Weeks since last non-XBB.1.5 dose, median (IQR)	59 (52-65)		98 (67-114)	NA
Case participants	33	10	290	323
Weeks since XBB.1.5 dose, median (IQR)	5 (3-8)		NA	NA
Weeks since last non-XBB.1.5 dose, median (IQR)	61 (54-65)		97.5 (68-110)	NA
Control participants	256	19	1081	1337
Weeks since XBB.1.5 dose, median (IQR)	5.5 (3-8)		NA	NA
Weeks since last non-XBB.1.5 dose, median (IQR)	58 (52-65)		98 (66-114)	NA
Vaccine effectiveness	%		95% CI	
Unadjusted[d]	52		29–67	
Adjusted[d,f]	54		31–70	
**Restricted to participants with previously-confirmed SARS-CoV-2 infection [b,g]**	**XBB.1.5 vaccinated[c]**		**Not XBB.1.5 vaccinated[c]**	**Total**
	**n**	**%**	**n**	**n**
Total participants	154	15	872	1026
Weeks since XBB.1.5 dose, median (IQR)	6 (3-8)		NA	NA
Weeks since last non-XBB.1.5 dose, median (IQR)	60 (53-67)		99 (69-116)	NA
Case participants	8	6	118	126
Weeks since XBB.1.5 dose, median (IQR)	4 (2.5-7)		NA	NA
Weeks since last non-XBB.1.5 dose, median (IQR)	61 (53-82.5)		97 (68-115)	NA
Control participants	146	16	754	900
Weeks since XBB.1.5 dose, median (IQR)	6 (3-8)		NA	NA
Weeks since last non-XBB.1.5 dose, median (IQR)	60 (53-67)		99 (70-117)	NA
Vaccine effectiveness	%		95% CI	
Unadjusted[d]	65		27–83	
Adjusted[d,f]	67		28–85	
**Excluding influenza cases from SARS-CoV-2 test-negative controls and restricted to participants with previously-confirmed SARS-CoV-2 infection [b,g]**	**XBB.1.5 vaccinated[c]**		**Not XBB.1.5 vaccinated[c]**	**Total**
	**n**	**%**	**n**	**n**
Total participants	127	16	654	781
Weeks since XBB.1.5 dose, median (IQR)	6 (3-8)		NA	NA
Weeks since last non-XBB.1.5 dose, median (IQR)	61 (53-66)		97 (66.5-115)	NA
Case participants	8	6	118	126
Weeks since XBB.1.5 dose, median (IQR)	4 (2.5-7)		NA	NA
Weeks since last non-XBB.1.5 dose, median (IQR)	61 (53-82.5)		97 (68-115)	NA
Control participants	119	18	536	655
Weeks since XBB.1.5 dose, median (IQR)	6 (3-8)		NA	NA
Weeks since last non-XBB.1.5 dose, median (IQR)	61 (53-66)		97 (64-115)	NA
Vaccine effectiveness	%		95% CI	
Unadjusted[d]	69		36–85	
Adjusted[d,f]	72		39–87	

BC: British Columbia; CI: confidence interval; IQR: interquartile range; NA: not applicable.

[a] Unlike primary analyses, excludes individuals with missing sex and comorbidity information.

[b] All COVID-19 analyses exclude participants <12 years and those who received a non-XBB.1.5 COVID-19 vaccine ≤24 before provincial launch of the publicly funded XBB.1.5 vaccine campaign (i.e., receipt of non-XBB.1.5 vaccine on or after 26 April 2023 in BC, 15 May 2023 in Ontario, and 25 April 2023 in Quebec).

[c] Vaccination status based upon provincial immunization registry data from BC, Ontario, and Quebec. Participants vaccinated < 2 weeks before acute respiratory illness onset or with unknown vaccination status or timing were excluded.

[d] VE was calculated as 1 − odds ratios (OR) × 100%. ORs compared test positivity between vaccinated and unvaccinated participants by logistic regression with covariate adjustment as specified.

[e] Adjusted for age group (12-49, 50-64, 65-79, ≥ 80 years), province (BC, Ontario, Quebec), calendar time (single epi-weeks 44-2) and, additionally, sex (male, female) and comorbidity (yes, no).

[f] Adjusted as per primary analysis for age group (12-49, 50-64, 65-79, ≥ 80 years), province (BC, Ontario, Quebec), and calendar time (single epi-weeks 44-2). Additional adjustment for sex and comorbidity (excluding participants with unknown data) did not alter point estimates by more than 2% (absolute) [not displayed].

[g] Restricted to participants with one or more previously confirmed SARS-CoV-2 infections (confirmed by nucleic acid amplification test (NAAT) or rapid antigen test (RAT)), as reported by participant or guardian. NAAT testing for SARS-CoV-2 was broadly available in Canada until early 2022 when self-administered RATs became widely accessible through private purchase and/or publicly funded access.

Supplementary Table S7. Age-stratified XBB.1.5 vaccine effectiveness estimates against COVID-19 among participants 12-64 years and ≥ 65 years in BC, Ontario and Quebec, Canadian Sentinel Practitioner Surveillance Network (SPSN), 29 October (week 44) 2023 – 13 January (week 2) 2024

	12 to 64 years				≥ 65 years			
**Without sex and comorbidity adjustment[a]**	XBB.1.5 Vaccinated[b]		Not XBB.1.5 Vaccinated[b]	Total	XBB.1.5 Vaccinated[b]		Not XBB.1.5 Vaccinated[b]	Total
	n	%	n	N	n	%	n	N
Total participants	176	10	1,506	1,682	166	39	264	430
Weeks since XBB.1.5 dose, median (IQR)	5 (3–8)		NA	NA	5 (3–7)		NA	NA
Weeks since last non-XBB.1.5 dose, median (IQR)	60 (54–65)		99 (83–117)	NA	57 (51–65)		75.5 (56–101)	NA
Case participants	15	6	239	254	18	26	51	69
Weeks since XBB.1.5 dose, median (IQR)	5 (3–9)		NA	NA	4.5 (3–7)		NA	NA
Weeks since last non-XBB.1.5 dose, median (IQR)	60 (55–68)		98.5 (75–115)	NA	61 (52–62)		81.5 (59–100)	NA
Control participants	161	11	1,267	1,428	148	41	213	361
Weeks since XBB.1.5 dose, median (IQR)	5 (4–8)		NA	NA	5.5 (3–8)		NA	NA
Weeks since last non-XBB.1.5 dose, median (IQR)	60 (54–65)		100 (84–118)	NA	57 (49–65)		73.5 (56–101)	NA
Vaccine effectiveness	%		95% CI		%		95% CI	
Unadjusted[c]	51		15–71		49		10–71	
Adjusted[c,d]	47		7–70		52		10–74	
**With sex and comorbidity adjustment[a,e]**	XBB.1.5 Vaccinated[b]		Not XBB.1.5 Vaccinated[b]	Total	XBB.1.5 Vaccinated[b]		Not XBB.1.5 Vaccinated[b]	Total
	n	%	n	N	n	%	n	N
Total participants	150	10	1,335	1,485	145	38	241	386
Weeks since XBB.1.5 dose, median (IQR)	6 (3–8)		NA	NA	5 (3–7)		NA	NA
Weeks since last non-XBB.1.5 dose, median (IQR)	60 (54–64)		99 (83–118)	NA	57 (49–65)		76 (57–101)	NA
Case participants	14	6	220	234	17	27	46	63
Weeks since XBB.1.5 dose, median (IQR)	6 (3–9)		NA	NA	4 (3–7)		NA	NA
Weeks since last non-XBB.1.5 dose, median (IQR)	60.5 (55–68)		98 (74–115)	NA	61 (52–62)		80 (60–99)	NA
Control participants	136	11	1,115	1,251	128	40	195	323
Weeks since XBB.1.5 dose, median (IQR)	6 (3–8)		NA	NA	5 (3–8)		NA	NA
Weeks since last non-XBB.1.5 dose, median (IQR)	60 (54–64)		99 (84–118)	NA	57 (48.5–65.5)		75 (56–101)	NA
Vaccine effectiveness	%		95% CI		%		95% CI	
Unadjusted[c]	48		8–70		44		-3 to 69	
Adjusted[c,f]	46		2–70		46		-3 to 72	

BC: British Columbia; CI: confidence interval; IQR: interquartile range; NA: not applicable.

[a] All COVID-19 analyses exclude participants <12 years and those who received a non-XBB.1.5 COVID-19 vaccine ≤24 before provincial launch of the publicly funded XBB.1.5 vaccine campaign (i.e., receipt of non-XBB.1.5 vaccine on or after 26 April 2023 in BC, 15 May 2023 in Ontario, and 25 April 2023 in Quebec).

[b] Vaccination status based upon provincial immunization registry data from BC, Ontario, and Quebec. Participants vaccinated < 2 weeks before acute respiratory illness onset or with unknown vaccination status or timing were excluded.

[c] VE was calculated as 1 − odds ratios (OR) × 100%. ORs compared test positivity between vaccinated and unvaccinated participants by logistic regression with covariate adjustment as specified.

[d] Adjusted for age group (12-49, 50-64, 65-79, ≥ 80 years), province (BC, Ontario, Quebec), calendar time (single epi-weeks 44-2).

[e] Unlike primary analyses, excludes individuals with missing sex and comorbidity information.

[f] Adjusted for age group (12-49, 50-64, 65-79, ≥ 80 years), province (BC, Ontario, Quebec), calendar time (single epi-weeks 44-2) and, additionally, sex (male, female) and comorbidity (yes, no).

References, Supplementary Material

1. Shu Y, McCauley J. GISAID: Global initiative on sharing all influenza data - from vision to reality. Euro Surveill. 2017;22(13):30494. https://doi.org/10.2807/1560- 7917.ES.2017.22.13.30494 PMID: 28382917
2. European Centre for Disease Prevention and Control (ECDC). Influenza virus characterization, Summary report, Europe, December 2023. Stockholm: ECDC [Accessed: 17 January 2024]. Available from: https://www.ecdc.europa.eu/en/publications-data/influenza-virus-characterization-summary-europe-december-2023
3. Aksamentov I, Roemer C, Hodcroft EB, Neher RA. Nextclade: clade assignment, mutation calling and quality control for viral genomes. Journal of Open Source Software. 2021;6(677). doi: https://doi.org/10.21105/joss.03773.
4. Freed N, Murphy L, Schwessinger B, Silander O. "Midnight" SARS-CoV2 genome sequencing protocol using 1200bp amplicon primer set v2 and the Nanopore Rapid library kit. Protocolsio. 2023. doi: https://dx.doi.org/10.17504/protocols.io.14egn2q2yg5d/v1.
5. Hickman R, Nguyen J, Lee TD, Tyson JR, Azana R, Tsang F, et al. Rapid, High-Throughput, Cost Effective Whole Genome Sequencing of SARS-CoV-2 Using a Condensed One Hour Library Preparation of the Illumina DNA Prep Kit. medRxiv. 2022:2022.02.07.22269672. doi: 10.1101/2022.02.07.22269672.
6. ARTIC Network. 2023. SARS-CoV-2 version 5.3.2 Scheme Release. Online Resource. [Accessed: 31 January 2024]. Available online at: https://community.artic.network/t/sars-cov-2-version-5-3-2-scheme-release/462.
7. Quick, J. 2020. nCOV-2019 Sequencing Protocol. Online Resource. [Accessed: 31 January 2024]. Available online at: https://www.protocols.io/view/ncov-2019-sequencing-protocol-bbmuik6w.pdf.
8. Illumina COVIDSeq Test Instructions for Use. California: Illumina; 2021. [Accessed: 31 January 2024]. Available from: https://support.illumina.com/content/dam/illumina-support/documents/documentation/chemistry_documentation/Illumina-COVIDSeq-Test/illumina-covidseq-test-instructions-for-use-canada-200004493-00.pdf
9. Bourgey M, Dali R, Eveleigh et al. GenPipes: an open-source framework for distributed and scalable genomic analyses. *GigaScience* 2019;8(6):giz037. [Accessed: 31 January 2024]. Available from: https://academic.oup.com/gigascience/article/8/6/giz037/5513895
10. Public Health Ontario (PHO). SARS-CoV-2 Genomic Surveillance in Ontario, January 22, 2024 Toronto: PHO; 2024 [26 January 2024]. Available from: https://www.publichealthontario.ca/-/media/Documents/nCoV/epi/covid-19-sars-cov2-whole-genome-sequencing-epi-summary.pdf?rev=41fb65ae9aa04fa78c67fd69ffb7c9f7&sc_lang=en.
11. Institut national de santé publique du Québec (INSPQ). Données sur les variants du SRAS-CoV-2 au Québec Wolfe: INSPQ; 2024 [Accessed: 26 January 2024]. Available from: https://www.inspq.qc.ca/en/node/26253.
12. British Columbia Centre for Disease Control (BCCDC). Respiratory Genomic Surveillance Vancouver: BCCDC; 2024 [Accessed: 26 January 2024]. Available from: https://bccdc.shinyapps.io/genomic_surveillance/.
13. Rambaut A, Holmes EC, O'Toole Á, Hill V, McCrone JT, Ruis C, et al. A dynamic nomenclature proposal for SARS-CoV-2 lineages to assist genomic epidemiology Nat Microbiol 2020;5:1403-07 DOI:10.1038/s41564-020-0770-5
14. O'Toole Á, Scher E, Underwood A, Jackson B, Hill V, McCrone JT, et al. Assignment of epidemiological lineages in an emerging pandemic using the pangolin tool. Virus Evolution. 2021;7(2):veab064. doi: 10.1093/ve/veab064.
15. De Serres G, Skowronski DM, Wu XW, Ambrose CS. The test-negative design: validity, accuracy and precision of vaccine efficacy estimates compared to the gold standard of randomised placebo-controlled clinical trials Euro Surveill. 2013; 18(37): pii=20585. https://doi.org/10.2807/1560-7917.ES2013.18.37.20585
16. Doll MK, Pettigrew SM, Ma J, Verma A. Effects of confounding bias in coronavirus disease 2019 (COVID-19) and influenza vaccine effectiveness test-negative designs due to correlated influenza and COVID-19 vaccination behaviors. Clin Infect Dis 2022;75(1):e564-e71. doi: 10.1093/cid/ciac234.
